# CrgA Protein Represses AlkB2 Monooxygenase and Regulates the Degradation of Medium-to-Long-Chain *n*-Alkanes in *Pseudomonas aeruginosa* SJTD-1

**DOI:** 10.3389/fmicb.2019.00400

**Published:** 2019-03-12

**Authors:** Nannan Ji, Xiuli Wang, Chong Yin, Wanli Peng, Rubing Liang

**Affiliations:** State Key Laboratory of Microbial Metabolism, School of Life Sciences and Biotechnology, Shanghai Jiao Tong University, Shanghai, China

**Keywords:** CrgA, AlkB2 monooxygenase, medium-to-long-chain *n*-alkane, repressor, imperfect mirror repeats (IIRs) structure

## Abstract

AlkB monooxygenases in bacteria are responsible for the hydroxylation of medium- and long-chain *n*-alkanes. In this study, one CrgA protein of *Pseudomonas aeruginosa* SJTD-1, a member of LysR family, was proved to regulate AlkB2 monooxygenase and the degradation of medium-to-long-chain *n*-alkanes (C_14_–C_20_) by directly binding to the upstream of *alkB*2 gene. Two specific sites for CrgA binding were found in the promoter region of *alkB*2 gene, and the imperfect mirror repeat (IIR) structure was proved critical for CrgA recognition and binding. Hexadecyl CoA and octadecyl CoA could effectively release the CrgA binding and start the transcription of *alkB2* gene, implying a positive regulation of metabolic intermediate. In the presence of medium-to-long-chain *n*-alkanes (C_14_–C_20_), deletion of *crgA* gene could enhance the transcription and expression of AlkB2 monooxygenase significantly; and in n-octadecane culture, strain S1_ΔalkB*1&*crgA_ grew more vigorously than strain S1*_ΔalkB*1&*crgA_*. Almost no regulation of CrgA protein was observed to *alkB*1 gene *in vitro* and *in vivo*. Therefore, CrgA acted as a negative regulator for the medium-to-long-chain *n*-alkane utilization in *P. aeruginosa* SJTD-1. The work will promote the regulation mechanism study of *n*-alkane degradation in bacteria and help the bioremediation method development for petroleum pollution.

## Introduction

Petroleum pollution is one of the most serious environmental problems caused by spilling or leakage of oil storage tanks, pipeline terminals or oil refineries (Alvarez and Vogel, 1991). The chronic oil pollutants usually persist in the environment over a long period of time and affect the ecosystem seriously. Biodegradation is one of the primary mechanisms to remediate the petroleum pollutants because of its high efficiency, low cost, and little secondary pollution, in which oleophilic microbes are used for elimination of hydrocarbon contaminants from environment (Das and Chandran, 2011; Macaulay and Rees, 2014). Microorganisms such as bacteria, fungi, algae have been reported for their ability to degrade hydrocarbon pollutants (Abbasian et al., 2015; Varjani, 2017). Bacteria are reported as the primary degraders and the most active agents in petroleum pollutant degradation (Abbasian et al., 2015). Bacterial spp. of genera *Alcanivorax, Thalassolituus, Oleiphilus, Pseudomonas, Dietzia, Amycolicicoccus, Acinetobacter, Rhodococcus, Burkholderia, Geobacillus, Gordonia, Bacillus*, and *Oleispira*, have been reported as hydrocarbon degraders (Whyte et al., 1999; Yuste et al., 2000; Golyshin et al., 2002; Hara et al., 2003; Kotani et al., 2003; Yakimov et al., 2003, 2004; Liu and Shao, 2005; Throne-Holst et al., 2006; Wang et al., 2006; Feng et al., 2007; Bødtker et al., 2009; Jung et al., 2010; Wang et al., 2010; Cai et al., 2011; Wang et al., 2011; Liu et al., 2012; Sun et al., 2012; Varjani and Upasani, 2016). Although their genetic backgrounds and degradation characteristics of these *n*-alkane-degrading microorganisms varied, all of them can response to the petroleum pollutants, and induce their cellular pathways for uptake, transportation, and carbon metabolism (Laczi et al., 2015; Liu et al., 2015; Wang et al., 2017).

The *n-*alkanes are degraded by terminal or sub-terminal oxidation; alkane hydroxylation catalyzed by monooxygenases/hydroxylases is considered as the first and critical step for *n*-alkane degradation in aerobic bacteria (Rojo, 2009). Three-class hydroxylases are considered responsible for the terminal hydroxylation of different-length *n-*alkanes and convert alkane to alkanols (Wang and Shao, 2013; Abbasian et al., 2015). The soluble non-heme di-iron monooxygenases (SDIMOs) and membrane-bound particulate copper-containing enzymes (pMMOs) are the main enzymes oxidizing the short-chain-alkanes (C_1_–C_5_) (Coleman et al., 2006; Cappelletti et al., 2015). The cytochrome P_450_ enzymes are responsible for the oxidization of medium-chain (C_6_–C_12_) *n*-alkanes (van Beilen et al., 2006). The integral-membrane alkane (AlkB) monooxygenases catalyzes the terminal oxidation of *n-*alkanes from medium-chain to long-chain (C_6_–C_20_) (Rojo, 2009; Liu et al., 2014; Parthipan et al., 2017). Moreover, LadA monooxygenase and the flavin-binding monooxygenase AlmA have been identified as the sub-terminal oxidation enzymes for long-chain *n-*alkanes (>C_20_) (Feng et al., 2007; Wang and Shao, 2012; Liu et al., 2018). The substrate spectrums of these alkane utilization enzymes usually overlap, and each strain always has two or three classes of monooxygenases to maintain efficient hydroxylation alkanes of different length (van Beilen and Funhoff, 2007; Varjani, 2017).

Meanwhile, microorganisms respond to and utilize *n-*alkanes by changing their cellular regulatory network and starting the transcription of related hydroxylase genes (Sabirova et al., 2006; Liu et al., 2015). Several regulators have been reported to regulate the utilization of *n*-alkanes in different bacteria, such as LuxR/MalT, AraC/XylS, TetR, GntR, BmoR, and AlmR (Wang and Shao, 2013; Wang et al., 2014; Liang et al., 2015, 2016). The AlkR of AraC/XylS family regulated the expression of the *alkM* gene in *Acinetobacter* sp. ADP1; BmoR (a σ54-dependent regulator) could activate the butane monooxygenase (BMO) to oxidize C_2_-C_8_ *n-*alkanes in *P. butanovora* (Ratajczak et al., 1998; Kurth et al., 2008). In *P. oleovorans* and *P. putida* GPo1, AlkS was induced by C_5_–C_10_ alkanes and then activated AlkB monooxygenase (Eggink et al., 1988; Yuste and Rojo, 2001). AlmR repressed the expression of AlmA monooxygenase and regulated the long-chain alkane metabolism in *A. dieselolei* (Wang and Shao, 2014). CypR (AraC family) and AlkX (TetR family) were identified as the activator and repressor of the alkane hydroxylases CYP153 and AlkW1 in *Dietzia* sp. (Liang et al., 2015, 2016). As multiple enzymes are involved in the biodegradation of *n-*alkanes in different-length, there must exist various transcriptional regulators and diverse transcription modes in different microorganisms. However, the participant regulators and their functional mechanisms are still not very clear.

Previously *P. aeruginosa* SJTD-1 (CGMCC No. 6584) has been isolated and identified capable of degrading medium- and long-chain *n*-alkanes (C_12_–C_32_); AlkB2 monooxygenase has been confirmed to be the major enzyme responsible for the hydroxylation of medium-to-long-chain *n-*alkanes (Liu et al., 2012, 2014). Proteomics analysis showed that many proteins responsible for uptake, transportation, carbon metabolism and regulation in *P. aeruginosa* SJTD-1 were changed for *n-*alkane response and utilization (Liu et al., 2015; Zhou et al., 2017). To study the regulation network for the medium-to-long-chain *n-*alkanes metabolism in this strain, the *in vivo* and *in vitro* experiments were carried out. A CrgA regulator was identified and its role in regulation of AlkB2 monooxygenase was studied in this work. Its binding mode, specific binding sites and conserved structure were characterized and its possible regulatory mechanism was explored.

## Materials and Methods

### Strains, Chemicals, Cultures, Enzymes and Oligonucleotides

All strains and plasmids used in this study were listed in Table 1. *n-*Dodecane, *n-*tetradecane, *n-*hexadecane, *n-*octadecane, *n-*pentadecane, *n-*eicosane, *n-*docosane, *n-*tetracosane, *n-*triacontane, *n-*hexane and their derivates (of HPLC grade, >99.0%) were purchased from Sigma-Aldrich (St. Louis, MO, United States). All other reagents were of analytical reagent grade. Luria-Bertani (LB) medium (10.0 g/L tryptone, 5.0 g/L yeast extract, 10.0 g/L NaCl, pH 7.0) and the minimal medium (BSM) [4.5 g/L KH_2_PO_4_, 13.75 g/L K_2_HPO_4_⋅3H_2_O, 2.0 g/L (NH_4_)_2_SO_4_, 0.16 g/L MgSO_4_⋅7H_2_O, 5.0 μg/L FeSO_4_, 1.0 μg/L CaCl_2_⋅2H_2_O, 2.0 μg/L MnCl_2_⋅4H_2_O, pH 7.4] were used in this study. The alkane-hexane solutions were prepared by dissolving the *n-*alkanes into the *n*-hexane (v/v or g/v) to 500 mg/mL and were supplied into the minimal medium to obtain various concentrations. The *n-*hexane was neither toxic nor utilized by strain *P. aeruginosa* SJTD-1 (Liu et al., 2014). All the enzymes for DNA-operation (DNA polymerases, T4 DNA ligases, restriction endonucleases and etc.) were purchased from TaKaRa Co. (Dalian, China) and the DNase I was the product of Thermo Fisher Scientific Inc. (Waltham, MA, United States). Oligonucleotides used for gene amplification, plasmid construction, reverse transcription (RT), quantitative PCR (q-PCR), homologous recombination, and electrophoretic mobility shift assay (EMSA) in this work were all synthesized by Invitrogen Ltd. (Shanghai, China) and listed in Table 2.

**Table 1:** Strains and plasmids used in this study.

Name	Description	Sources/reference
**Strains**		
*P. aeruginosa* SJTD-1	Strain with the alkane-degradation capability isolated from oil-contaminated soil, Wild-Type	Liu et al., 2012
*E. coli* DH5α	*F′*/*endA1 hsdR17* (*rK-mK-*) *glnV44 thi-1 recA1 gyrA* (*NalR*) *relA1 Δ* (*lacIZYA-argF*)*U169 deoR* (*Φ80dlacΔ* (*lacZ*)*M15*)	Invitrogen
*E. coli* BL21(DE3)	Protein allogeneic expression strains	Novagen
*E. coli* SM10	*thi-1 thr leu tonA lacY supE recA::RP4-2-Tc::Mu*, Km^R^	De Lorenzo, 1994
S1*_ΔalkB*1*_*	*ΔalkB*1 mutant of *P. aeruginosa* SJTD-1	Liu et al., 2014
S1*_ΔalkB*2*_*	*ΔalkB*2 mutant of *P. aeruginosa* SJTD-1	Liu et al., 2014
S1*_ΔcrgA_*	*ΔcrgA* mutant of *P. aeruginosa* SJTD-1	This study
S1*_ΔalkB2*&*crgA_*	*ΔalkB*2 *ΔcrgA* double-knockout mutant of *P. aeruginosa* SJTD-1	This study
S1*_ΔalkB*1&*crgA_*	*ΔalkB*1 *ΔcrgA* double-knockout mutant of *P. aeruginosa* SJTD-1	This study
**Plasmids**		
pET28a	*E. coli* expression plasmid, Km^r^	Novagen
pET28a-crgA	Plasmid pET28a inserted with *crgA* gene at *Bam* HI/*Nde* I sites, Km^r^	This study
pEX18Gm	*oriT*^+^ *sacB*^+^, gene replacement vector with MCS from pUC18, Gm^r^	Hoang et al., 1998
pEX-crgA-UD	Plasmid pEX18Gm containing the 500 bp upstream fragment and 500 bp downstream fragment of *crgA* gene, Gm^r^	This study
pBSPPc-Gm	*oriT*^+^gene replacement vector derived from pBR322, Ap^r^, Gm^r^	Xu et al., 2013
pBS-eGFP	*egfp* gene inserted into plasmid pBSPPc-Gm without promoter, Ap^r^, Gm^r^	This study
pBS-P*_alkB*2*_*-eGFP	*egfp* gene inserted into plasmid pBSPPc-Gm under the promoter of *alkB*2 gene, Ap^r^, Gm^r^	This study


**Table 2:** Oligonucleotides used in this study.

Name	Sequence(5′-3′)^∗^
**Cloning primers**	
CrgA-orf-F	ggggggCATATGAGCCTGCGCCTTGAAG
CrgA-orf-R	ggggggGGATCCTGAGCGAGGCTCAGCGCA
eGFP-F	gggcccAAGCTTATGGTGAGCAAGGGCGAGGAG
eGFP-R	gggcccGGATCCTTAGTACAGCTCGTCCATGCCGAGA
PalkB2-UF	gggcccATCGATACGTCATGGTGATCCTTTTATC
PalkB2-UR	gggcccAAGCTTGGGAAGTCCTCGTATTTATCTTG
**Homologous recombination primers**	
CrgA-UF	ggggggGAGCTCTCCTCCAGCGCCGAGAGG
CrgA-UR	ggggggGGATCCTTCAAGGCGCAGGCTCATGG
CrgA-DF	ggggggGGATCCCAGTTTCTCCAGCAGGCCCT
CrgA-DR	ggggggCTGCAGTACAGCGCCCTGTGCATGG
**RT-qPCR primers**	
16sRNA-QF	TCGCCTTGGTAGGCCTTTAC
16sRNA-QR	GGGAGCTTGCTCCTGGATTC
AlkB2-QF	AGGAAGCCAGCGAAGTGCC
AlkB2-QR	TCGTGGGAGACGGTGATGC
CrgA-QF	GCCTGCGCCTTGAAGACATA
CrgA-QR	ACTTGTAGAAGAAGCCGCCG
**EMSA assay primers**	
AlkB2-187bp-FAM-UR	FAM-GGGAAGTCCTCGTATTTATCTTGTT
AlkB2-187bp-UF	ACGTCATGGTGATCCTTTTATCC
AlkB2-46bp-FAM-UR	FAM-GGGAAGTCCTCGTATTTATCTTGTTAGATTGTCTGACAATTGTCCT
AlkB2-46bp-UF	AGGACAATTGTCAGACAATCTAACAAGATAAATACGAGGACTTCCC
AlkB2-b1-70bp-FAM-UR	FAM-GGGAAGTCCTCGTATTTATCTCCCTTGGCCGGCCTGCCCCATCCCTGTTAGATTGTCTGACAATTGTCCT
AlkB2-b1-70bp-UF	AGGACAATTGTCAGACAATCTAACAGGGATGGGGCAGGCCGGCCAAGGGAGATAAATACGAGGACTTCCC
AlkB2-b2-63bp-FAM-UR	FAM-GGGAAGTCCTCGTATTTATCTTCGTTCTGTCCGCCGCTTGTTAGATTGTCTGACAATTGTCCT
AlkB2-b2-63bp-UF	AGGACAATTGTCAGACAATCTAACAAGCGGCGGACAGAACGAAGATAAATACGAGGACTTCCC
AlkB2-b3-70bp-FAM-UR	FAM-GACAATTGTCCTCCCTTGGCCGGCCTGCCCCATCCCAACCTTCGTTCTGTCCGCCGCTTCGCCGGGAAGC
AlkB2-b3-70bp-UF	GCTTCCCGGCGAAGCGGCGGACAGAACGAAGGTTGGGATGGGGCAGGCCGGCCAAGGGAGGACAATTGTC
AlkB2-b4-58bp-FAM-UR	FAM-GGGAAGTCCTCGTATTTATCTCCCTTGGCCGGCTGTTAGATTGTCTGACAATTGTCCT
AlkB2-b4-58bp-UF	AGGACAATTGTCAGACAATCTAACAGCCGGCCAAGGGAGATAAATACGAGGACTTCCC
AlkB2-b5-55bp-FAM-UR	FAM-GGGAAGTCCTCGTATTTATCTTCGTTCTGTTGTTAGATTGTCTGACAATTGTCCT
AlkB2-b5-55bp-UF	AGGACAATTGTCAGACAATCTAACAACAGAACGAAGATAAATACGAGGACTTCCC
AlkB2-b6-70bp-FAM-UR	FAM-GGGAAGTCCTCGTATTTATCTCCCTTGGCCGGCCCCTACCCCGTCTGTTAGATTGTCTGACAATTGTCCT
AlkB2-b6-70bp-UF	AGGACAATTGTCAGACAATCTAACAGACGGGGTAGGGGCCGGCCAAGGGAGATAAATACGAGGACTTCCC
AlkB2-b7-63bp-FAM-UR	FAM-GGGAAGTCCTCGTATTTATCTTCGTTCTGTTCGCCGCCTGTTAGATTGTCTGACAATTGTCCT
AlkB2-b7-63bp-UF	AGGACAATTGTCAGACAATCTAACAGGCGGCGAACAGAACGAAGATAAATACGAGGACTTCCC


*^∗^The uppercase letters mean the pairing bases, the lowercase letters mean protecting bases and the underline means the restriction enzyme sites.*

### Standard DNA Manipulation

The PCR procedure was performed at 95°C for 5 min, 30 cycles of 95°C for 1 min, 55°C for 1 min, and 72°C for 1.5 min, and 72°C for 5 min. All the plasmids were constructed by ligating the restriction-enzyme-treated PCR fragments and plasmid bones with T4 DNA ligase. Chemical transformation and the electroporation-mediated transformation were used for the plasmids transformation into *E. coli* strains and *P. aeruginosa* strains, respectively. All constructed plasmids were confirmed by DNA sequencing in Invitrogen Ltd. (Shanghai, China). The recovery of PCR fragment, extraction of plasmid DNA, and isolation of genome DNA were achieved by respective kits from TIANGEN Co. (Beijing, China) and following the corresponding protocols. RT and q-PCR were performed using the PrimeScript Reverse Transcriptase kit and Probe qPCR kit (TaKaRa, Dalian, China). Other general techniques for the agarose gel electrophoresis, native polyacrylamide gel electrophoresis (PAGE) and sodium dodecyl sulfate-polyacrylamide gel electrophoresis (SDS–PAGE) were carried out with standard protocols.

### Pull-Down Assay and Mass Spectrometry Analysis

*Pseudomonas aeruginosa* SJTD-1 was cultured in 1 L BSM medium with 500 mg/L *n-*octadecane as the carbon source at 37°C to OD_600_ about 1.0. Cells were harvested by centrifugation at 8,000 rpm for 10 min at 4°C and re-suspended in 40 mL ice-cold lysis buffer [20 mM Tris-HCl, 300 mM NaCl, 10 mM imidazole, 5 mM β-Mercaptoethanol (β-ME), 1 mM phenylmethanesulfonyl fluoride (PMSF), 10% glycerol, pH 7.9]. Crude extracts was obtained by sonication on ice and centrifugation at 12,000 rpm for 30 min at 4°C. Then the clarified solution was mixed into the streptavidin-coated magnetic particles with the immobilized DNA fragment, the 500 bp upstream fragment of *alkB*2 gene. Proteins that bound to the target DNA in magnetic particles were isolated and detected by mass spectrometry. Mass spectral data were collected in +ESI mode in separate runs on a Waters HDMS-QTOF synapt GI mass spectrometer operated in a scan mode of 50–500 m/z. The predicted composition was calculated with MASCOT software.

### Multiple Sequences Alignment, Phylogenetic Tree Construction and Structure-Homology Modeling

The multiple sequences alignments (MSA) of DNAs and proteins were performed using DNAMAN software. The phylogenetic tree of CrgA homologues was constructed based on their gene sequences using MEGA 7.0 software in the neighbor-joining method with 1,000 replications. The structure-homology modeling of the CrgA protein (from *P. aeruginosa* SJTD-1) was performed in SWISS-MODEL with the CrgA protein (3HHG) of *Neisseria meningitidis* as a template.

### Heterogenous Expression and Affinity Purification of Recombinant CrgA Protein

The recombinant CrgA protein was expressed in *E. coli* BL21 (DE3) strain and purified by affinity chromatography. Briefly, *E. coli* BL21 (DE3) cells containing plasmid pET28a-crgA was inoculated in 10 mL LB with 50 μg/mL kanamycin and cultured at 37°C. The overnight cultures were transferred into fresh LB with 50 μg/mL kanamycin and cultured to about 0.5 OD_600_. 0.5 mM isopropyl β-D-1-thiogalactopyranoside (IPTG) was added and 3 h induction at 37°C was performed. Cells were harvested by centrifugation at 8,000 rpm for 10 min at 4°C and re-suspended in 25 mL ice-cold lysis buffer (20 mM Tris-HCl, 300 mM NaCl, 10 mM imidazole, 5 mM β-ME, 1 mM PMSF, 10% glycerol, pH 7.9). Cell disruption was performed with sonication on ice, and the cell lysate was clarified by centrifugation at 12,000 rpm and 4°C for 30 min. The cell supernatants were loaded into the Ni-NTA resin (Bio-Rad, Hercules, CA, United States) at 4°C and washed with washing buffer (20 mM Tris-HCl, 300 mM NaCl, 5 mM β-ME, 10% glycerol, 1 mM PMSF, 20 mM or 50 mM imidazole, pH 7.9). Finally, the recombinant protein was eluted from the column using elution buffer (20 mM Tris-HCl, 300 mM NaCl, 5 mM β-ME, 10% glycerol, 1 mM PMSF, 250 mM imidazole, pH 7.9). All the eluted fractions were analyzed with 15% SDS-PAGE followed by staining with Coomassie Brilliant Blue R250 (Sigma-Aldrich, St. Louis, MO, United States). The eluted recombinant CrgA protein was dialyzed and stored in storage buffer (20 mM Tris-HCl, 50 mM NaCl, 1 mM EDTA, 1 mM DTT, 50% glycerol, pH 8.0) at -80°C.

### Homologous Recombination and Construction of crgA-Knockout Mutants

The *crgA* gene was knocked out using the two-step homologous recombination method (Hoang et al., 1998). The pEX-crgA-UD plasmid was constructed by inserting the 500 bp upstream and 500 bp downstream fragments of *crgA* gene into plasmid pEX18Gm (Hoang et al., 1998). Then plasmid pEX-crgA-UD was transformed into *E. coli* SM10 strain before the conjugation with *P. aeruginosa* SJTD-1. Fifty μg/mL gentamycin and 25 μg/mL tetracycline were used for the first-step selection; 10% sucrose was used to induce the second-step recombination and generate the *crgA*-deleted mutant. The deletion of *crgA* gene was performed in the wild-type strain SJTD-1, the *alkB*1-knockout strain S1*_ΔalkB*1*_* and the *alkB*2-knockout strain S1*_ΔalkB*2*_* (Liu et al., 2014); the resulting strains were named S1*_ΔcrgA_*, S1*_ΔalkB*1&*crgA_*, S1*_ΔalkB*2&*crgA_* respectively (Table 1).

### Cell Growth Detection of Wild-Type Strain and Mutant Strains

The cell growth of wild-type strain SJTD-1 and all the knockout mutants (S1_ΔalkB*1*_, S1_ΔalkB*2*_, S1_ΔcrgA_, S1_ΔalkB*1&*crgA_, S1_ΔalkB*2&*crgA_) were determined with *n*-alkanes as the sole carbon source (Liu et al., 2014). Single colony was inoculated into 10 mL LB broth and cultured overnight at 37°C. The harvested cells were washed thrice with sterilized water and re-suspended in BSM medium to OD_600_ about 2.0. Ten μL cell pellet was inoculated into the wells of a 10 × 10 multi-well plate, each containing 190 μL BSM medium with different concentrations of *n*-alkanes (C_12_–C_24_). The initial OD_600_ of each well was 0.1. Wells containing cells without *n-*alkanes and wells containing *n-*alkanes without cells were used as blank controls. The 10 × 10 multi-well plates were loaded onto an Automatic Growth Curve Analyzer (BioScreen Testing Service, Inc., Torrance, CA, United States), and cultured at 180 r/min for 7 days at 37°C. Cell densities were determined by detecting the OD_600_ every hour. At least five independent experiments (three paralleled samples in each experiment) were conducted and the average values were calculated with standard errors. Statistical analysis were performed with the cell densitities at three time points (96, 120, and 140 h) using SPSS 24 for *t*-test calculation and the *P-*value was calculated (*P*-value < 0.05, significant).

### Electrophoretic Mobility Shift Assay (EMSA) Detection of CrgA to DNA Fragments

DNA fragments of different length were amplified from the upstream regions of *alkB*2 gene using different oligonucleotides. The long fragments (>100 bp) were amplified with primers labeled with FAM at 5′-site, and the short fragments (<100 bp) were obtained by annealing two paired oligonucleotides (Table 2). The two oligonucleotides of equimolar concentrations (10 μM) were mixed in the annealing buffer (10 mM Tris-HCl, 50 mM KAc, 1 mM EDTA, pH 8.0), incubated at 95°C for 5 min, and then cooled slowly to room temperature. The EMSA detection was performed by mixing CrgA protein with the labeled DNA fragments at different molecular ratios and incubating in the binding buffer (20 mM Tris-HCl, 50 mM NaCl, 1 mM DTT, 0.1 mM EDTA, pH 7.5) at 37°C for 30 min. All input DNA amounts were 4 pmol in the 20 μL binding system. The mixture of the binding assay was analyzed with 8% native PAGE and visualization using the BioRad Imaging System (Bio-Rad, Hercules, CA, United States). The EMSA detection of CrgA protein to the upstream fragment of *alkB*1 gene and the mutant fragments of *alkB*2 gene (alkB2-b1 to alkB2-b7) were also performed.

### Determination of the *n*-Alkane Metabolites Effect on CrgA Binding

Different metabolites of *n-*hexadecane and *n-*octadecane (hexadecanol, octadecanol, octadecanoic acid, palmitic acid, sodium palmitate, sodium stearate, hexadecyl coenzyme A, and octadecyl coenzyme A) were used to determine their effects on the binding of CrgA to the upstream region of *alkB*2 gene. Ethyl acetate and DMSO were used as controls. The binding of CrgA to the three fragments of *alkB*2 gene (alkB2-b1, alkB2-b2, alkB2-b3) was performed same as above. Then different metabolites of 20, 100, or 500 pmol were added into the 20 μL reaction system, followed with 15-min incubation at 37°C. The mixture was analyzed with 8% native PAGE and visualization by the BioRad Imaging System (Bio-Rad, Hercules, CA, United States).

### DNase I Foot-Printing Assay and Sequence Analysis of the CrgA Binding Sites

Four hundred ng DNA fragments (alkB2-U187) amplified from the upstream region of *alkB*2 gene was bound with CrgA protein at 25°C for 30 min same as above. Then 0.015 U DNase I was added into the binding system with incubation at 25°C for 1 min. The reaction products were extracted with phenol to remove proteins, and ethanol was added to precipitate DNA fragments. The precipitated DNA fragment was dissolved in ultra-pure water and sequenced. The DNA fragment (alkB2-U187) with BSA protein and the DNA fragment without binding ability to CrgA protein (alkB2-U46) were used as controls.

### RT-qPCR

The wild-type strain and the mutant strains were inoculated in BSM medium with glucose or various alkanes (C_12_–C_24_) and cultured to the mid-exponential phase. Total RNA was extracted with Total RNA Extraction Reagents (Vazyme, Nanjing, China) according to the protocol. The yield of RNA was estimated using a Nanodrop UV spectrometer (Thermo Fisher Scientific, Waltham, MA, United States). DNase treatment was used to remove the possible genomic DNA contamination. RT was performed with approximately 1 μg RNA and 20 ng random primers using PrimeScript Reverse Transcriptase Kit (TaKaRa, Dalian, China). q-PCR was achieved using Premix Ex Taq (Probe qPCR) (TaKaRa, Dalian, China) and the gene-specific primers in an IQTM 5 Multicolor Real-time PCR Detection System (Bio-Rad, Hercules, CA, United States). The conditions were set at 95°C for 3 min, and 40 cycles of 95°C for 10 s, 57°C for 30 s, and 72°C for 30 s; a final melting analysis was performed by slow heating with 10 s increments of 0.5°C from 57°C to 95°C. The one without reverse transcriptase was used as negative control. The threshold cycle (Cq) value of each sample was determined during the exponential phase of amplification, and the relative fold change in mRNA quantity was calculated using the DDCt method (Livak and Schmittgen, 2001). At least five independent experiments (three paralleled samples in each experiment) were conducted for each sample, and the average values were calculated with standard errors. Statistical analysis were performed with the relative mRNA quantities using SPSS 24 for *t*-test calculation and the *P-*value was calculated (*P*-value < 0.05, significant).

### Expression Detection of Green Fluorescent Protein (GFP) in Different Strains

Green fluorescent protein fluorescence assays was performed to detect the promoter activity and expression level of *alkB*2 gene in different strains. The *egfp* gene was cloned into pBSPPc-Gm to generate plasmid pBS-eGFP (Table 1). Then the upstream promoter region of *alkB*2 gene was inserted the upstream of *egfp* gene to form the plasmid pBS-P*_alkB*2*_*-eGFP. The two plasmids were transformed into the wild-type strain and the *crgA*-knockout mutant strains. All the recombinant strains were cultured in BSM medium with 0.4% glucose or 500 mg/L *n-*alkanes of different lengths. Cells were collected at different time points to measure GFP fluorescence with excitation at 485 nm and emission at 527 nm. At least five independent experiments were performed, and the average values were calculated with standard errors. Statistical analysis were performed with the relative GFP fluorescence using SPSS 24 for *t*-test calculation and the *P* value was calculated (*P*-value < 0.05, significant).

## Results

### CrgA Protein of *P. aeruginosa* SJTD-1 Was a Member of LysR-Type Transcriptional Regulators (LTTRs)

**FIGURE 1:**
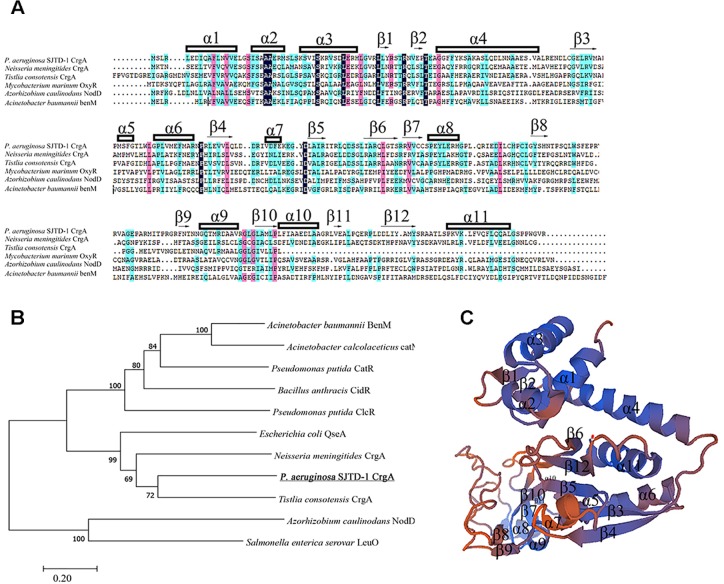
Multiple sequences alignment, evolutionary analysis and structure prediction of CrgA in *P. aeruginosa* SJTD-1. **(A)** Multiple sequences alignment of different CrgA homologues including the CrgA of *P. aeruginosa* SJTD-1 (ANI09566), CrgA of *N. meningitides* (AAF37819.1), CrgA of *Tistlia consotensis* (WP_085121968.1), OxyR of *Mycobacterium marinum* (WP_020729003.1), NodD of *Azorhizobium caulinodans* (AAA26190.1), and BenM of *A. baumannii* (WP_000423280.1). The α-helix and β-sheet were numbered and the conserved amino acids were marked. **(B)** The phylogenetic tree of CrgA in *P. aeruginosa* SJTD-1 and other LTTR regulators. The Kimura two-parameter distance model was used and bootstrap analysis was performed with 1,000 repetitions. **(C)** The predicted structure of CrgA in *P. aeruginosa* SJTD-1. The structure model was constructed by the homology-modeling method with CrgA of *N. meningitides* (3HHG) as the template.

AlkB2 monooxygenase was proved to be the major enzyme hydroxylating the medium-to-long-chain *n-*alkanes (C_14_–C_20_) in strain *P. aeruginosa* SJTD-1 (Liu et al., 2014). To find its potential regulators, the pull-down assay and mass spectrometry were performed. Results showed that one 34 KD protein was identified because of its specific binding to the upstream region of *alkB*2 gene, which was predicted to be a CrgA-like protein. This CrgA-like protein (ANI09566) of *P. aeruginosa* SJTD-1 contained 304 amino acids, encoded by gene *crgA* (CP015877.1: 3086790-3087704), far from the *alkB*2 gene (CP015877.1: 3423611-3424744). Secondary structure analysis of this CrgA protein showed that there were 11 α-helixes and 12 β-strands in it; three α-helixes (α1 to α3) and two β-strands (β1 to β2) were in its N-terminal region, forming a conserved winged-helix-turn-helix (w-HTH) structure and functioning as the DNA binding domain. Its C-terminal region containing seven α-helixes (α5 to α11) and ten β-strands (β3 to β12) were highly variable and responsible for the effector binding (Figure 1A). Evolutionary analysis revealed this CrgA-like protein was close to the CrgA protein of *Neisseria meningitides* (Figure 1B). Tertiary structure prediction showed that their spatial structures were similar; the N-terminal region and C-terminal region were connected through a long α-helix (α4) structure (Figure 1C). Therefore, this identified CrgA-like regulator was a member of the CrgA sub-family of LTTRs.

### CrgA Protein Bound to the Specific Imperfect Mirror Repeat (IIR) Site in the Promoter Region of *alkB2* Gene

**FIGURE 2:**
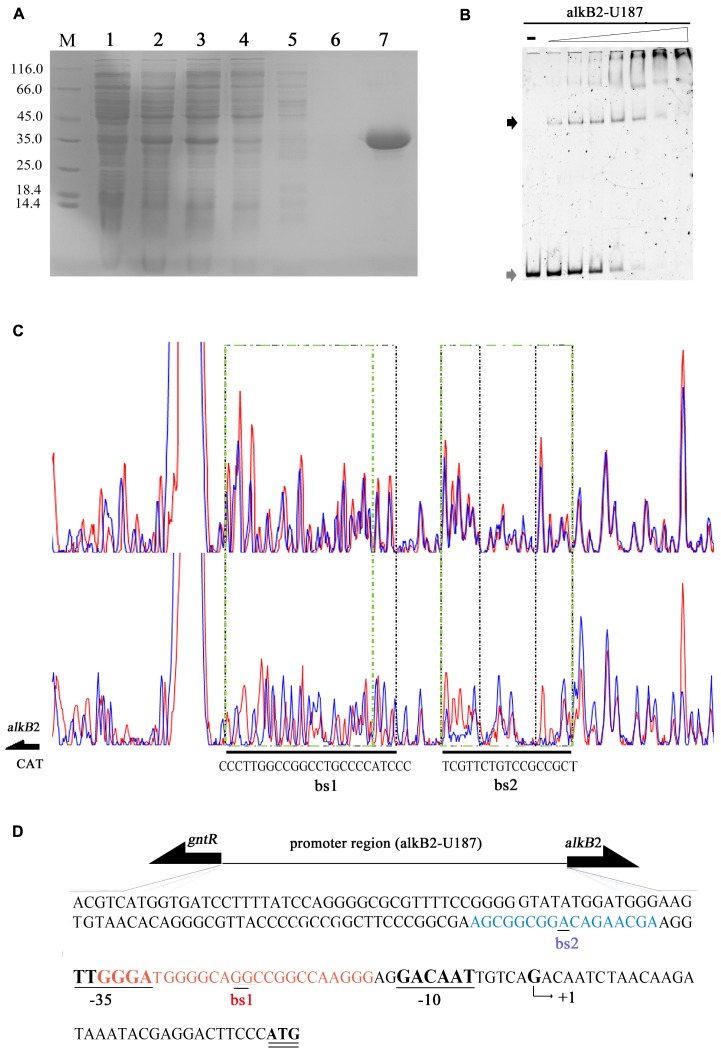
Affinity purification and binding detection of the recombinant CrgA protein to the upstream fragment of *alkB*2 gene. **(A)** The SDS-PAGE image of recombinant CrgA protein. M, protein marker (Thermo Fisher Scientific, 14.4 KD–116.0 KD); Lane 1, cell lysis solution before induction; lane 2, cell lysis solution after induction; lane 3, the supernatant of lysed cells; lane 4, the effluent solution; lane 5, solution of first wash; lane 6, solution of last wash and lane 7 the eluted protein solution. **(B)** Native PAGE image of EMSA assay with the recombinant CrgA protein and the 5′-FAM labeled 187 bp fragments of *alkB*2 gene (alkB2-U187). The input of alkB2-U187 fragments in each sample was 4 pmol, and the molar ratios of CrgA/alkB2-U187 fragment ranged from 0, 2:1, 4:1, 8:1, 16:1, 32:1, 64:1, and 128:1. The lane marked dash meant the free DNA fragment without protein. The band of free DNA fragments were marked with gray arrow and the conjunct DNA were marked with black arrow. **(C)** The sequencing profiles of DNase I foot-printing assay performed with CrgA protein and the 5′-FAM labeled 187 bp fragments of *alkB*2 gene (alkB2-U187). The upper one was the sequence map of the fragment alkB2-U187 without CrgA protein after DNase I digestion; and the lower one was the sequence map of fragment alkB2-U187 binding with CrgA protein in 8:1 molar ratios (protein/DNA) after DNase I digestion. The protected regions were lined with green dashed frame, and marked as bs1 and bs2. **(D)** Sequence of the upstream region of *alkB*2 gene. The two CrgA binding site was showed in red font (bs1) and blue font (bs2). The arrow represented the predicted transcriptional start site (TSS, +1), and -10 (GACAAT) and -35 (TTGGGA) regions were underlined.

The recombinant CrgA protein was obtained by heterologous expression and affinity purification, with a yield of 12 mg/L and purity of 98% (Figure 2A). EMSA detection showed that the CrgA protein could bind to the 187 bp upstream fragment of *alkB*2 gene (alkB2-U187) in a concentration-dependent manner. No dissociation was observed over the 32:1 protein-DNA ratio (Figure 2B). However, very weak binding ability and low specificity was observed when this CrgA protein mixed with the upstream region of *alkB*1 gene, implying that the regulation preference of this CrgA protein to AlkB2 monooxygenase (Supplementary Figure S1A).

The DNase I foot printing assay showed that CrgA protein bound to the upstream fragment of *alkB*2 gene at two specific sites, bs1 and bs2 (Figure 2C). No binding was observed when BSA used (Supplementary Figure S2A). Interestingly, the structures of the two binding sites were mirror like, similar to the imperfect mirror repeat (IIR) structure. For the bs1 site, its sequence was GGGA-N3-GGC-N-GG-N-CGG-N3-AGGG, using the middle GG bases for symmetry; for the bs2 site, the central base A was used for symmetry and the sequence was AGC-N5-A-N5-CGA. The IIR structure in bs1 was more stringent than that in bs2 (Figure 2C). Moreover, the bs2 site was close to the predicted -35 region of *alkB*2 gene; the bs1 site overlapped the -35 region and located between the -35 and -10 region of *alkB*2 gene (Figure 2D). No obvious mirror structure was found in the upstream region of *alkB*1 gene (Supplementary Figure S1B). These results indicated CrgA could bind to the specific sites in the promoter region of *alkB*2 gene and may influence the transcription of AlkB2 monooxygenase.

**FIGURE 3:**
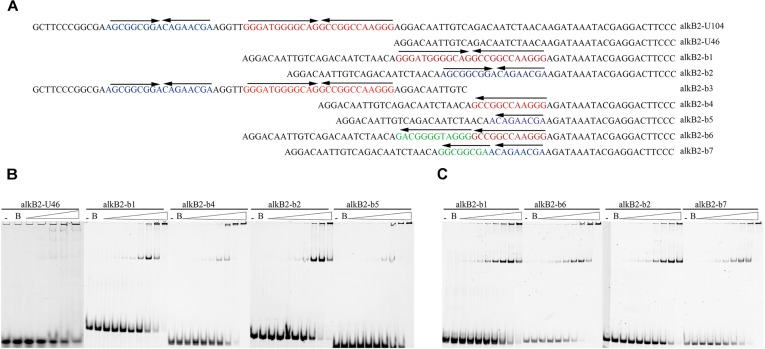
EMSA detection for the binding of CrgA protein to different DNA fragments. **(A)** Sequences of different DNA fragments for EMSA detection. The upper arrows of each oligonucleotides meant the direction of IIR structures. The alkB2-U104 was the sequences of the upstream 104 bp region of *alkB*2 gene. Fragment alkB2-U46 was the upstream 46 bp fragment of *alkB*2 gene without CrgA binding ability and was used for the insertion of different binding sites. The fragments alkB2-b1 and alkB2-b2 contained the full IIR structure of one binding site, bs1 or bs2; the fragment alkB2-b3 contained the two binding sites. The fragments alkB2-b4 and alkB2-b5 had only half of IIR structure of one binding site, bs1 or bs2. The destroyed IIR structure (reversed sequences) of bs1 or bs2, were in the fragments alkB2-b6 and alkB2-b7. **(B)** Native PAGE image of EMSA assay with CrgA protein to the fragments with IIR structure. The fragments alkB2-U46, alkB2-b1, alkB2-b2, alkB2-b4 and alkB2-b5 were used. **(C)** Native PAGE image of EMSA assay with CrgA protein to fragments with destroyed IIR structure. The fragmentsalkB2-b1, alkB2-b2, alkB2-b6 and alkB2-b7 were used. The input fragments in each sample were 4 pmol and the molar ratios of CrgA/DNA fragment ranged from 0.5:1, 1:1, 2:1, 4:1, 8:1, 16:1, 32:1, and 64:1 (from left to right). The lane marked dash meant the free DNA fragment without protein. The lane marked B was the DNA fragment mixed with BSA protein in 64:1 molar ratio.

To study the structure specificity of the CrgA-binding sites, several mutant DNA fragments containing the intact, partial or destroyed mirror structure were constructed and used for EMSA detection. The 46 bp upstream region of *alkB*2 gene (alkB2-U46), which was confirmed not binding to CrgA protein, was selected as template fragment to insert different binding sequences (Supplementary Figure S2B). The IIR structure was maintained in the mutant fragments (b1/b2), and only half of IIR structure was reserved in the mutant fragments (b4/b5). The destroyed IIR structure was generated by changing the opposite direction of the mirror structure into identical direction (b6/b7) (Figure 3A). EMSA results showed that the binding of CrgA protein to the fragments containing half of the IIR structure (b4/b5) or no IIR structure (b6/b7), were much weaker than that to the fragments with full IIR structure (b1/b2); half of the IIR structure probably acted as the basic binding unit (Figure 3B,C). Therefore, these results demonstrated that the IIR structure and its integrity were crucial for the efficient binding of CrgA to the promoter region of *alkB*2 gene.

### Specific Binding of CrgA to the alkB2 Promoter Could Be Released by Long-Chain Fatty Acyl-CoA

**FIGURE 4:**
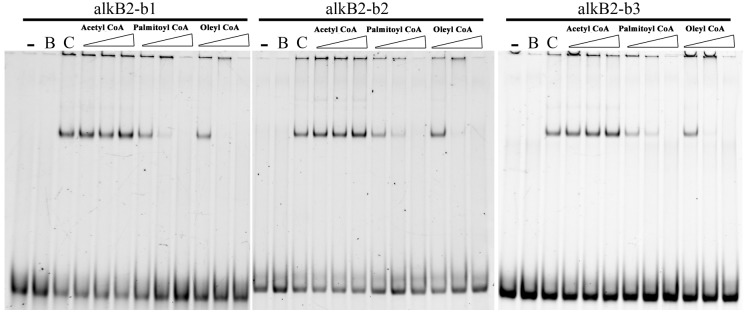
EMSA detection of the alkane metabolites effect on the CrgA binding to different DNA fragments. The fragments of alkB2-b1, alkB2-b2, and alkB2-b3 were used for the target DNA fragments for CrgA protein binding. Acetyl coenzyme A, palmitoyl coenzyme A and oleyl coenzyme A were added into the binding system in 20, 100, and 500 pmol (from left to right). The lane with dash meant the blank control of target DNA fragments. The lane marked B meant the target DNA mixed with BSA protein in 8:1 ratio. The lane marked C was the target DNA mixed with CrgA protein in 8:1 ratio. The input of DNA fragments in each sample was 4 pmol.

Effects of *n-*alkanes and their metabolites on the specific binding of CrgA to the promoter region of *alkB*2 were analyzed. Addition of hexadecyl coenzyme A and octadecyl coenzyme A into the binding system could efficiently release the specific binding of CrgA to the fragments containing the two binding sites (bs1 and bs2), dependent on the chemical’s concentration. No sequence preference was observed and no release was found with acetyl coenzyme A (Figure 4). Although palmitic acid and octadecanoic acid had little influence on the interaction, other derivatives of *n-*alkanes could not release CrgA binding from the upstream fragments of *alkB*2 (Supplementary Figure S3). These results indicated that long-chain fatty acyl-CoA (C_16_–C_18_) could influence the specific binding of CrgA to the target region of *alkB*2 gene, probably through a positive feedback of intermediates.

### CrgA Repressed the Expression of AlkB2 Monooxygenase

**FIGURE 5:**
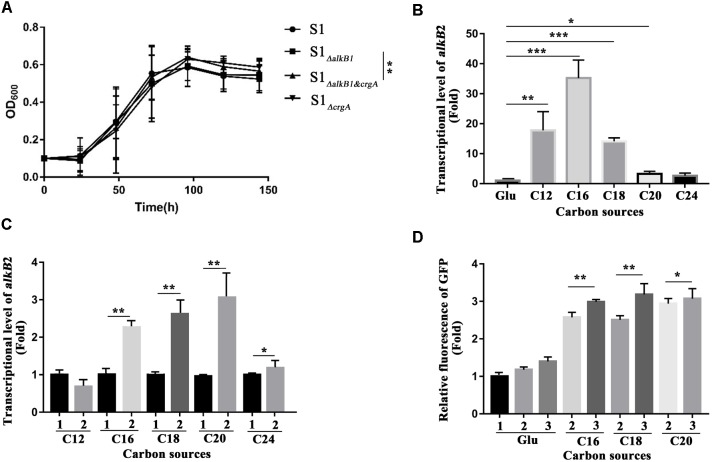
Cell growth detection, the transcription and expression analysis of *alkB*2 gene in wild-type strain and mutant strains. **(A)** The growth curves of different strains cultured with *n-*octadecane as sole carbon source. Wide type SJTD-1 strain (S1, •), the *alkB*1-knockout strain (S1 Δ_alkB*1*_, 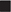), the *crgA*-knockout strain (S1 Δ*_crgA_*, 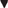), and the *alkB*1/*crgA* double knockout strain (S1*Δ_alkB*1&*crgA_*, 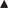) were detected. The concentration of C_18_ was 500 mg/L and the initial concentration of strains was OD_600_ = 0.1. At least five independent experiments (three paralleled samples in each experiment) were conducted and the average values were calculated with standard errors. The statistical analysis were performed with the cell numbers of strains at three time points (96, 120, and 144 h) and the *P* value was marked with star (^∗^*P-*value < 0.1; ^∗∗^*P-*value < 0.05; ^∗∗∗^*P-*value < 0.01). **(B)** The transcriptional levels of *alkB*2 gene in strain SJTD-1 cultured with glucose and different alkanes (C_12_–C_24_). The transcriptional level of *alkB*2 gene in glucose (Glu) was set as 1.0. At least five independent experiments (three paralleled samples in each experiment) were conducted and the average values were calculated with standard errors. The statistical analysis were performed with the transcriptional levels of *alkB*2 gene in strain SJTD-1 cultured with glucose and different same *n-*alkane, and marked with star (^∗^*P-*value < 0.1; ^∗∗^*P-*value < 0.05; ^∗∗∗^*P-*value < 0.01). **(C)** The transcriptional levels of *alkB*2 gene in the wild-type strain SJTD-1 and strain S1*_ΔcrgA_* cultured with different alkanes (C_12_–C_24_). 1 represented strain SJTD-1, and 2 represented strain S1*_ΔcrgA_*. The transcriptional levels of *alkB*2 gene in strain SJTD-1 cultured with the corresponding *n*-alkanes was set as 1.0, respectively. At least five independent experiments (three paralleled samples in each experiment) were conducted and the average values were calculated with standard errors. The statistical analysis was performed with the transcriptional levels of *alkB*2 gene in strain S1*_ΔcrgA_ and* strain SJTD-1 cultured with same *n-*alkane, and marked with star (^∗^*P-*value < 0.1; ^∗^*P-*value < 0.05; ^∗∗∗^*P-*value < 0.01). **(D)** The fluorescence of *egfp* gene under the promoter of *alkB*2 gene in strain SJTD-1 and strain S1*_ΔcrgA_* cultured with glucose and different alkanes (C_16_–C_20_). 1 was strain SJTD-1 containing plasmid pBS-eGFP; 2 was strain SJTD-1 containing plasmid pBS-P_alkB*2*_-eGFP; 3 was strain S1*_ΔcrgA_* containing plasmid pBS-P_alkB*2*_-eGFP. The GFP fluorescence in strain SJTD-1 with plasmid pBS-eGFP cultured with glucose was set as 1.0. At least five independent experiments (three paralleled samples in each experiment) were conducted and the average values were calculated with standard errors. The statistical analysis were performed with the GFP fluorescence in strain SJTD-1 containing plasmid pBS-P_alkB*2*_-eGFP (2) and that in strain S1*_ΔcrgA_* containing plasmid pBS-P_alkB*2*_-eGFP (3) cultured with same *n-*alkane, and marked with star (^∗^*P-*value < 0.1; ^∗∗^*P-*value < 0.05; ^∗∗∗^*P-*value < 0.01).

The regulation of CrgA to AlkB2 monooxygenase and *n-*alkane utilization was analyzed by detecting the cell growth and the transcription and expression of *alkB*2 gene. For cell growth analysis, at least five independent experiments (three paralleled samples in each experiment) were conducted at each time point, and the average cell densitities at the stationary phase were performed the statistical analysis using SPSS 24 for *t*-test calculation between every two strains. Results indicated that in the C_18_-alkane condition, strain S1*_ΔalkB*1*_* grew normally, while strain S1*_ΔalkB*2*_* grew slower than the wild-type strain, consistent with previous report (Supplementary Figure S4) (Liu et al., 2014). However, at the stationary phase, the growth difference of the three strains (SJTD-1, S1*_ΔalkB*1*_* and S1*_ΔalkB*2*_*) was not statistically significant. Notably, the growth diversity in strains S1*_ΔalkB*1*_* and S1*_ΔalkB1*&*crgA_* was significant at the stationary phase, and the cell densitity of strain S1*_ΔalkB1*&*crgA_* was higher than that of strain S1*_ΔalkB*1*_* (Figure 5A and Supplementary Figure S5). Beside, the growth difference was not statistically significant between the strains S1*_ΔalkB*2*_* and S1*_ΔalkB2*&*crgA_* (Supplementary Figure S4). These results implied that *crgA*-knockout could not siginificantly influence the cell growth; while the deletion of *crgA* gene in the *alkB1*-knockout strain (S1*_ΔalkB1*&*crgA_*/S1*_ΔalkB*1*_*) could improve the cell growth, in which *alkB2* gene was the major gene for the utilization of medium-to-long chain *n*-alkane. At the meanwhile, the growth difference was not statistically significant when the *crgA* gene was knocked out in the *alkB2*-knockout strain (S1*_ΔalkB2*&*crgA_*/S1*_ΔalkB*2*_*). It indicated that CrgA was probably involved in the regulation of AlkB2, not AlkB1. As the growth difference was not so big, it was much likely that other alkane hydroxylases and other transcriptional regulators also participated in utilization of medium-to-long-chain *n-*alkanes in strain SJTD-1, although the AlkB monooxygenases played important roles (Liu et al., 2014).

Transcriptional levels of *alkB*2 gene in strains SJTD-1 and S1*_ΔcrgA_* cultured with different carbon sources (glucose and C_12_–C_24_ alkanes) were analyzed. When strain SJTD-1 was cultured with C_12_, C_16_, and C_18_ alkanes, the transcription of *alkB*2 gene was significantly induced (over 10-fold); about 2–4 folds increase in the transcription of *alkB*2 gene was also observed in the culture with C_20_ and C_24_ alkanes (Figure 5B). The transcription of *alkB*2 gene could be further enhanced after deletion of *crgA*; in the C_16_–C_20_ alkanes culture, significant transcriptional promotion (over 2.4-fold) was observed compared to those in wild-type strain. However, no significant change was observed when strains cultured with C_12_ and C_24_ alkanes (Figure 5C). These results demonstrated that the transcription of *alkB*2 gene was repressed by CrgA and in the medium-to-long-chain alkane conditions, this repression was released to start *alkB*2 transcription. Furthermore, the expression detection of *egfp* gene under the promoter of *alkB*2 gene was performed in strains SJTD-1 and S1*_ΔcrgA_*. Although the GFP fluorescence changes in the two strains were not as significant as the transcription diversity, similar trends were also observed. The GFP fluorescence of strains cultured with C_16_, C_18_, and C_20_ alkanes were higher than those cultured in glucose; when cultured with medium-to-long-chain alkanes, the expression of *egfp* gene under the promoter of *alkB*2 gene in strain S1*_ΔcrgA_* was higher than that in strain SJTD-1 (Figure 5D). The inconsistency in the transcription and translation may due to differences in the expression of genome and plasmid or the influences of other regulators. Taken together, these results demonstrated that CrgA could repress the transcription of *alkB*2 gene and regulate the utilization of medium-to-long-chain *n-*alkane in strain SJTD-1.

## Discussion

Alkanes are the major component of crude oil, and thus alkane-degrading microorganisms are widely distributed in nature. In spite of their relative inertness, *n-*alkanes are degraded in the presence of oxygen and support the abundant growth of many different bacteria (Watkinson and Morgan, 1990). Based on the chain length of the aliphatic hydrocarbons utilized, *n-*alkane degrading microorganisms are classified into three groups: methanotrophs, gaseous alkane-utilizing (C_2_-C_4_) microorganisms and liquid alkane-catabolizing (C_5_–C_20_) microorganisms (Rojo, 2010). Many microorganisms have been reported capable of utilizing liquid *n-*alkanes of short-chain (<C_6_), medium-chain (C_6_–C_12_) and medium-to-long chain (C_14_–C_20_); some microorganisms have been found to be able to utilize the solid long-chain alkanes (>C_20_) (Rojo, 2009; Abbasian et al., 2015).

The hydrocarbon degradation in most aerobic bacteria include the initial degradation of alkane with oxidation of methyl group to form alcohol, which is then dehydrogenated via aldehyde to the corresponding carboxylic acid that can then be metabolized by β-oxidation. Oxidation of terminal methyl to form the primary alcohol by introducing molecular oxygen into hydrocarbon is the first step in this process, and the monooxygenase responsible for this reaction is the key enzyme for *n-*alkanes utilization (Abbasian et al., 2015). Based on the molecular structure and the supporting electron transport system, monooxygenases are classified into rubredoxin-dependent enzymes and cytochrome P450-containing monooxygenases (Rojo, 2009; Abbasian et al., 2015). The rubredoxin-dependent enzymes are composed of a rubredoxin reductase, a rubredoxin and an alkane hydroxylase. In most bacteria, the integral membrane non-heme di-iron monooxygenases of AlkB type are mainly used for the initial step (Ji et al., 2013). Two groups are distinguished by a single tryptophan residue that determines the length of the alkane substrates (van Beilen et al., 2005). The type-1 integral membrane alkane hydroxylases (AH1) oxidize short- and medium-chain alkanes (C_5_–C_10_), mainly found in pseudomonads and in some other gamma-Proteobacteria (like *Alcanivorax borkumensis*); while most members of AlkB enzyme belonging to AH2 group oxidize medium-to-long chain and long-chain alkanes (>C_12_) (van Beilen and Funhoff, 2007). The AlkB enzymes receive electrons from NADH through a mononuclear iron rubredoxin reductase and a di-nuclear iron rubredoxin, functioning in a complex; their encoding genes, the AH gene (*alkB*1 or *alkB*2), rubredoxin gene (*alkG*) and rubredoxin reductase gene (*alkT*) are often in one cluster (van Beilen et al., 2001). Bacteria always have several copies of alkane monooxygenase. In *Rhodococcus*, at least four alkane monooxygenase genes (*alkB*1, *alkB*2, *alkB*3 and *alkB*4) was found; the *alkB*1 and *alkB*2 genes were parts of cluster, whereas *alkB*3 and *alkB*4 were isolated genes (Whyte et al., 2002). In addition to the AlkB enzymes, cytochrome P450 alkane monooxygenase systems, the flavin-containing alkane dioxygenase and other two monooxygenase for long-chain alkanes, the soluble flavoprotein alkane monooxygenase LadA and the flavin-binding monooxygenase AlmA were discovered in different *n-*alkane-degrading strains (Throne-Holst et al., 2006; van Beilen et al., 2006; Feng et al., 2007). In fact, a combination of different alkane oxidation systems with overlapping substrate ranges exist in many *n*-alkane degraders for wide substrate spectrum (van Beilen and Funhoff, 2007; Varjani, 2017). For example, *A. borkumensis* isolates possess two AlkB and three CYP153 (Sabirova et al., 2006); two AlkB and one CYP153 gene were detected in *Dietzia* sp. DQ12-45-1b strain (Nie et al., 2014). Our previous work showed that the isolated *P. aeruginosa* SJTD-1 could utilize medium- and long-chain *n*-alkanes (C_12_–C_32_) as a sole carbon source; two AlkB monooxygenases (AlkB1/2), two P_450_ enzymes (P_450_-1/2), two AlmA (AlmA-1/2) and two LadA monooxygenases (LadA-1/2) was found in its genome (Liu et al., 2012). Sequence alignments showed the two AlkB monooxygenases belonged to AH1 and AH2 group; the *alkB*2 was in a cluster of xanthine dehydrogenase iron-sulfur gene (*xdh-FeS*) and a xanthine dehydrogenase Mo(II) binding gene (*xdh-MoII*). This AlkB1 monooxygenase hydroxylated short-to-medium chain alkanes (C_10_–C_16_) and AlkB2 monooxygenase mainly oxidized medium-to-long-chain alkanes (C_12_–C_24_), respectively (Liu et al., 2014).

At the same time, in petroleum environment, alkane-degrading microorganisms would change their regulatory network and initiate the alkane utilization (Sabirova et al., 2006; Liu et al., 2015). As AlkB enzymes play main role in the alkane hydroxylation, their regulation mechanisms are concerned. Several regulators have been reported involved in its regulation as activators or repressors, while the regulation of alkane biodegradation remains unclear as the multiple alkane monooxygenase systems and various regulation mode (Ratajczak et al., 1998; Kurth et al., 2008; Wang and Shao, 2014; Liang et al., 2015, 2016, 2017). In *P. aeruginosa* SJTD-1, the global response to alkane environments was observed with the significant change in the expression of many proteins involved in uptake, transportation, carbon metabolism and regulation (Liu et al., 2015; Zhou et al., 2017). However, the regulator and its function mechanism for *n-*alkane degradation in this strain was undetermined. In this work, we identified a CrgA protein regulated the utilization of medium-to-long-chain *n*-alkanes by repressing the expression of *alkB*2 gene directly. It bound to the specific sites in promoter region and the IIR structure was critical for protein recognition and binding. Hexadecyl coenzyme A and octadecyl coenzyme A, the alkane-degrading intermediates, could release CrgA protein from the promoter of *alkB*2 and start the *alkB*2 gene transcription, forming an intermediate-feedback regulation network. The CrgA regulator characterization in this work will advance the regulatory mechanism study in *n-*alkane-degraders and help the bioremediation process for petroleum pollution.

The identified CrgA protein in this work was found containing the conserved HTH domain and belonging to the LTTR family. LTTR regulators are the most abundant type of transcriptional regulator in prokaryotes, which regulate the expression of diverse genes involved in virulence, metabolism, quorum sensing, and motility. The LTTR regulators normally contain a conserved N-terminal HTH motif for DNA binding and a variable C-terminal region with a little conservation for inducer binding (Maddocks and Oyston, 2008). Their regulation are generally achieved through an LTTR box with the sequence T-N11-A at the binding site, varying in length and base pair composition (Maddocks and Oyston, 2008). Here the identified CrgA of *P. aeruginosa* SJTD-1 was similar to CrgA regulator found in *N. meningitides* (Deghmane et al., 2000). In *N. meningitidis*, CrgA participated in the regulation of pili/capsule synthesis (Matthias and Rest, 2014). It normally forms an octameric structure and binds in octamer form to the consensus sequence (T-N11-A) of the *crgA*/*mdaB* promoter region, acting as either a repressor or an activator (Ieva et al., 2005; Sainsbury et al., 2008, 2009). CrgA could also repress the transcription of several genes encoding surface-expressed proteins such as the type IV pili and capsule virulence factors to facilitate strong adhesion (Deghmane et al., 2000, 2002, 2004; Deghmane and Taha, 2003; Derkaoui et al., 2016). Moreover, CrgA protein was considered a key-factor for low osmolarity adaption in halotolerant *Rhodospirillaceae Tistlia consotensis* (Rubiano-Labrador et al., 2014). Therefore, all these findings in *N. meningitides, R. Tistlia consotensis*, and *P. aeruginosa S*JTD-1 (this work) indicated that CrgA played an important role in bacteria adaption to various environments and may be distributed among different bacteria.

In this work, CrgA was proved to regulate the expression of *alkB*2 gene through direct binding to the promoter region of *alkB*2 gene, and deletion of *crgA* gene could promote the cell growth. Notably, CrgA could not bind to the promoter region of *alkB*1 gene; further sequence alignment showed there were no specific binding site or mirror repeat structure in the upstream of *alkB*1 gene. The growth difference was not statistically significant between the *ΔalkB*2*ΔcrgA*-double-knockout strain (S1*_ΔalkB*2&*crgA_*) and the *ΔalkB*2*-*knockout strain (S1*_ΔalkB*2*_*); all these results implied that CrgA was probably not involved in the regulation of *alkB*1 gene. Interestingly, a slight interaction was observed when CrgA protein was mixed with the upstream fragments of other hydroxylases genes for long-chain-alkane utilization (*almA*-2, *ladA-*1, and *ladA-*2) (data not shown). And as the *alkB*2** gene was in a cluster of xanthine dehydrogenase iron-sulfur gene (*xdh-FeS*) and a xanthine dehydrogenase Mo (II) binding gene (*xdh-MoII*), CrgA might also regulate the transcription of other genes in operon with *alkB*2. Therefore, CrgA protein may globally regulate the alkane utilization of medium-to-long-chain and long-chain in *P. aeruginosa* SJTD-1; other regulatory proteins may also participate the regulation of AlkB1 and short-chain-alkane degradation. As multiple catabolic enzymes and complex network must exist in bacteria to adapt the stress environment caused by *n-*alkanes and keep efficient utilization, one enzyme may be likely regulated by several different regulators, and one regulator may also have multiple targets. This may be the reason that no significant difference in alkane utilization was observed in the *crgA*-knockout strain.

In this work, two specific regions (bs1 and bs2) were identified in the promoter of *alkB*2 gene which was important for the DNA binding of CrgA protein. A T-N14-A sequence was found in the bs1 region, while no sequence like an LTTR box was found in the bs2 region. In both of the bs1 and bs2 regions, the IIR structures were observed and were proved critical for the recognition and DNA binding of CrgA. Symmetric sequences such as palindromic sequences, direct repeats, reverse repeats or mirror repeats, are ubiquitous in the genome of eukaryotes and prokaryotes. These motifs can affect the transcription of nearby genes by acting as the recognition sites of the allosteric domain in regulators (Maletta et al., 2014; Oliver et al., 2016). In *Helicoverpa zea*, a GCT mirror repeat was found essential for the basal activity and flavone-induced luciferase activity of cytochrome *P450 CYP321A*1 promoter, and a TATA inverted repeat was necessary for its flavone-induced luciferase activity (Zhang et al., 2010). Palindromic motifs and mirror repeats on transposons could be recognized by transposase to facilitate the gene movement within genome (Bigot et al., 2005). In addition, NicR2, an HTH-containing transcriptional regulator critical for nicotine degradation in *P. putida* S16, could directly bind to a 28 bp IIR in the promoter region of *nic*2 gene with a putative tetramer and regulate the nicotine utilization (Wang et al., 2014). In this study, the medium-to-long-chain fatty acyl-CoA (hexadecyl coenzyme A and octadecyl coenzyme A) was proved able to release the binding of CrgA from the promoter of *alkB*2 gene. As aerobic alkane degradation before β-oxidation is achieved by oxygenase in four steps and generates the corresponding intermediates: fatty alcohols, fatty acids and fatty acid acyl-CoAs (Ji et al., 2013). It meant that the downstream intermediate could release the CrgA repressor and facilitate the transcription of AlkB2 monooxygenase. The release of this intermediate may due to the allosteric effect of CrgA caused by the effector binding (Bacik et al., 2015). Therefore, further work would be conducted to determine the structure of CrgA of *P. aeruginosa* SJTD-1 and the CrgA-DNA or CrgA-effector complex, and explore its recognition and interaction mechanism.

## Author Contributions

RL designed the experiments and wrote the manuscript. NJ and XW performed the experiments. CY and WP assisted the experiments. All the authors discussed the results and commented on the manuscript.

## Conflict of Interest Statement

The authors declare that the research was conducted in the absence of any commercial or financial relationships that could be construed as a potential conflict of interest.
