# Supplementary material for: CrgA Protein Represses AlkB2 Monooxygenase and Regulates the Degradation of Medium-to-Long-Chain n-Alkanes in Pseudomonas aeruginosa SJTD-1
Source: Front Microbiol. 2019 Mar 12;10:400. doi: 10.3389/fmicb.2019.00400 (PMC6422896; doi:10.3389/fmicb.2019.00400)
Supplement: Supplementary file 1 [file Data_Sheet_1.PDF]

Fig. S1. EMSA detection of CrgA protein to the upstream region of *alkB*1 gene. (A) Native PAGE image of EMSA assay with CrgA protein to the 5′-FAM labelled 450 bp and 200 bp upstream DNA fragments of *alkB*1 gene (alkB1-U450 and alkB1-U200). The input of DNA fragments in each sample was 4 pmol, and the molar ratios of CrgA protein/dsDNA fragment were ranging from 0, 8:1, 16:1, 32:1, 64:1, and 128:1 (from left to right). The lane marked dash meant the free DNA fragment without protein. The lane marked B was the DNA fragment mixed with BSA protein in 128:1 molar ratio. (B) The sequences of the upstream region of *alkB*1. The predicted transcriptional start site (+1), -10 region (GACAAT) and -35 (TTGGGA) region were underlined.

A

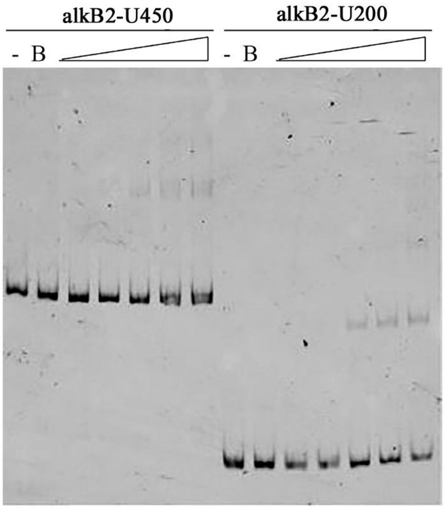

B

tccttgaccagttcgacgatcttctcctgggagacgggcagttgcttgtccagggcg
tagatggtgcggcggttcttgatggaggagaggaatgcgtcagacatcgtgaggc
tccttgtttggaaattggattcgatattagcgacggcctcgatgagatcaatgggaa
ataaccggtgattcctatcggctgatgcgatggatacccggcttccccaggaaagc
cgggtatccacggtccagcgcggccggcagcgagagacggccagtacggatgaa
aatggaatatcgagaaagtcgatgggcataaaaccgcgaggacttggcctttgatt
-35
caggtcaagcagggtgaaggcggggaaggcggccaacctttagtgatggtgacct
-10 +1
ggcagcccggctcggtgatggttcgattgtaggattgtcggacaaaaactggagcg
cacgATG
*alkB*1
