# Supplementary material for: CrgA Protein Represses AlkB2 Monooxygenase and Regulates the Degradation of Medium-to-Long-Chain n-Alkanes in Pseudomonas aeruginosa SJTD-1
Source: Front Microbiol. 2019 Mar 12;10:400. doi: 10.3389/fmicb.2019.00400 (PMC6422896; doi:10.3389/fmicb.2019.00400)

**Fig. S2 Sequencing profiles of DNase I foot-printing assay of CrgA protein to fragments alkB2-U187 and alkB2-U46.** (A). The sequence map of 5’-FAM labelled fragment alkB2-U187 mixed with BSA protein in 8:1 molar ratios (protein/DNA) after DNase I digestion. (B). The sequence map of 5’-FAM labelled fragment alkB2-U46 mixed with CrgA protein in 8:1 molar ratios (protein/DNA) after DNase I digestion.

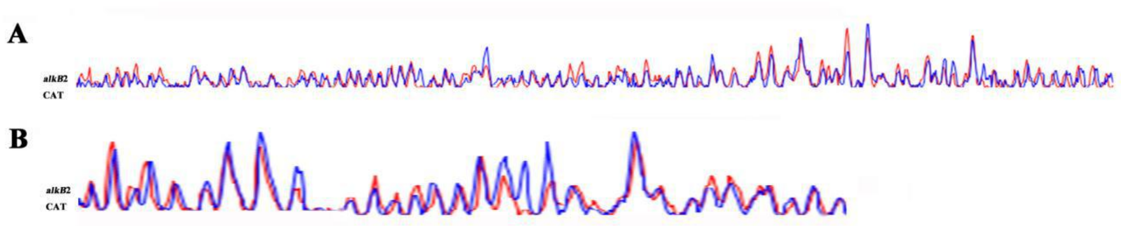

Supplement: Supplementary file 2 [file Data_Sheet_2.PDF]
