# Supplementary material for: CrgA Protein Represses AlkB2 Monooxygenase and Regulates the Degradation of Medium-to-Long-Chain n-Alkanes in Pseudomonas aeruginosa SJTD-1
Source: Front Microbiol. 2019 Mar 12;10:400. doi: 10.3389/fmicb.2019.00400 (PMC6422896; doi:10.3389/fmicb.2019.00400)

Fig. S3. Effect of *n*-alkane derivates on the binding of CrgA to the upstream DNA fragments of *alkB*2 gene. Ethyl acetate, palmitic acid, octadecanoic acid, sodium palmitate, sosium stearate, hexadecanol, octadecanol and DMSO were added into the binding system in 100 pmol, and 500 pmol (from left to right). Ethyl acetate and DMSO were used as controls. The lane with dash meant the blank control of fragment alkB2-b3. The lane marked B meant the fragment alkB2-b3 mixed with BSA protein in 8:1 ratio. The lane marked C was the fragment alkB2-b3 mixed with CrgA protein in 8:1 ratio. The input of DNA fragment in each sample was 4 pmol.

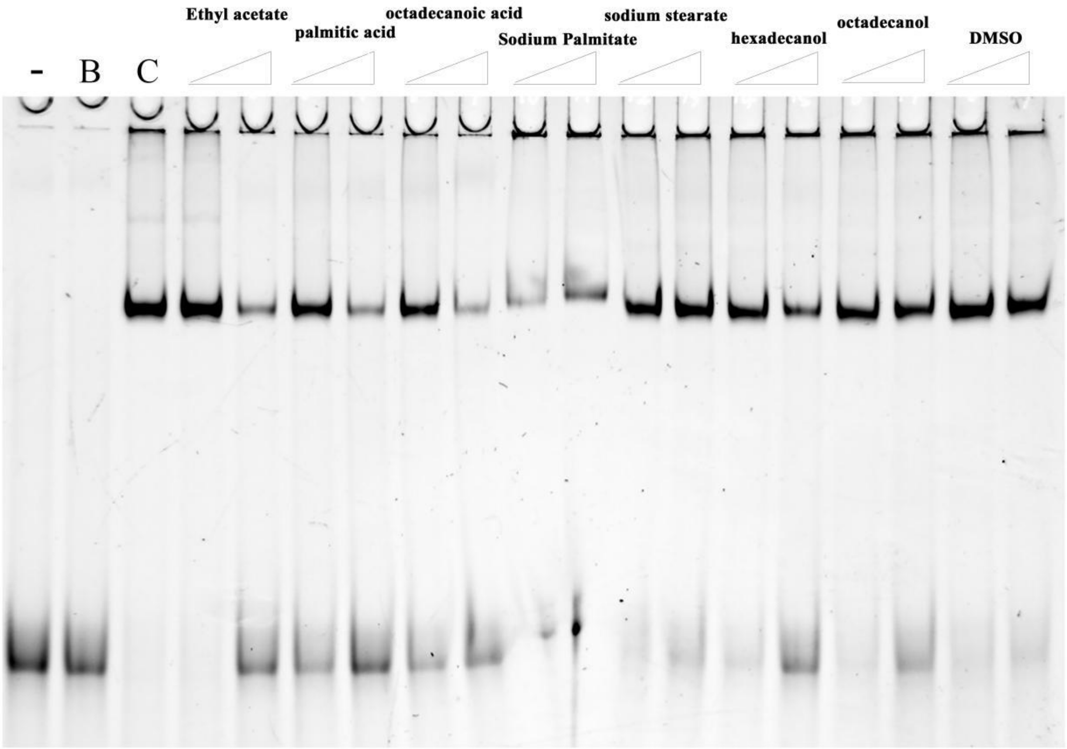

Supplement: Supplementary file 3 [file Data_Sheet_3.PDF]
