# Supplementary material for: CrgA Protein Represses AlkB2 Monooxygenase and Regulates the Degradation of Medium-to-Long-Chain n-Alkanes in Pseudomonas aeruginosa SJTD-1
Source: Front Microbiol. 2019 Mar 12;10:400. doi: 10.3389/fmicb.2019.00400 (PMC6422896; doi:10.3389/fmicb.2019.00400)

Fig. S4 Cell growth detection of wild type strain and mutant strains. Growth curves of different strains cultured with n-octadecane as sole carbon source. Wide type SJTD-1 strain (S1, ●), the *alkB*2-knockout strain ($S1_{\Delta alkB2}$, ■), the *crgA*-knockout strain ($S1_{\Delta crgA}$, ▼), and the *alkB*2/*crgA* double knockout strain ($S1_{\Delta alkB2\,\&\,crgA}$, ▲) were detected. The concentration of n-octadecane was 500 mg/L and the initial concentration of strains was OD_{600}=0.1. At least five independent experiments (three paralleled samples in each experiment) were conducted and the average values were calculated with standard errors. The statistical analysis were performed with the cell densities of strains at three time points (96 h, 120 h and 144 h) using SPSS 24 for *t*-test calculation, and the growth difference was not statistically significant.

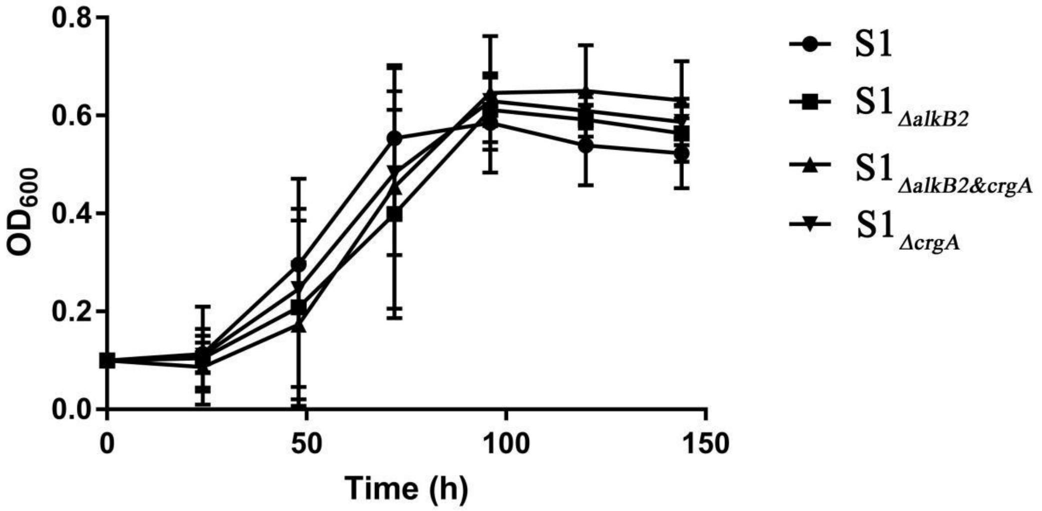

Supplement: Supplementary file 4 [file Data_Sheet_4.PDF]
