# Supplementary material for: CrgA Protein Represses AlkB2 Monooxygenase and Regulates the Degradation of Medium-to-Long-Chain n-Alkanes in Pseudomonas aeruginosa SJTD-1
Source: Front Microbiol. 2019 Mar 12;10:400. doi: 10.3389/fmicb.2019.00400 (PMC6422896; doi:10.3389/fmicb.2019.00400)

Fig. S5 Cell growth detection of mutant strains S1*ΔalkB2* and S1*ΔalkB2 & crgA*. Growth curves of the two mutant strains, the *alkB*1-knockout strain (S1*ΔalkB1*, ■) and the *alkB*1/*crgA* double knockout strain (S1*ΔalkB1 & crgA*, ▲), cultured with n-octadecane as sole carbon source. The concentration of n-octadecane was 500 mg/L and the initial concentration of strains was OD_{600}=0.1.

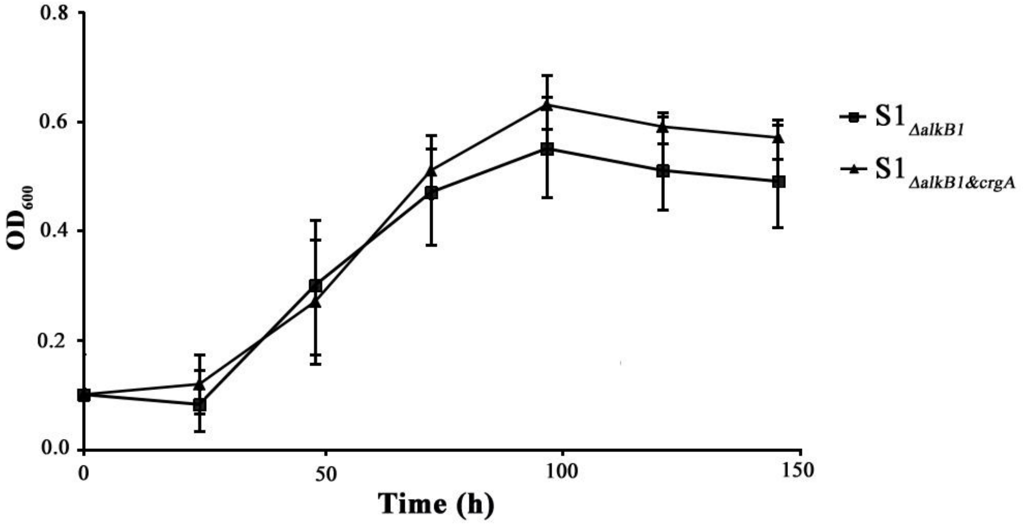

Supplement: Supplementary file 5 [file Data_Sheet_5.PDF]
